# Stability and changes in meaning in life profiles and their impact on mental health among chinese university students: a latent transition analysis

**DOI:** 10.3389/fpsyg.2025.1529851

**Published:** 2025-02-06

**Authors:** Sylvia Y. C. L. Kwok, Siqi Fang, Bella Meici Huang, Alebel Addis Tesfaw, Xi Deng

**Affiliations:** ^1^Department of Social and Behavioral Sciences, City University of Hong Kong, Kowloon, Hong Kong SAR, China; ^2^Department of Educational Psychology, The Chinese University of Hong Kong, Shatin, Hong Kong SAR, China; ^3^Department of Psychology, Wolkite University, Addis Ababa, Ethiopia

**Keywords:** meaning in life, mental health, anxiety, depression, latent transition analysis

## Abstract

**Background:**

Research on meaning in life (MIL) has predominately adopted variable-centered approaches. The few person-centered studies conducted were generally cross-sectional in nature and have failed to address changes in MIL. Furthermore, few studies have explored the stability of and changes in MIL on wellbeing.

**Methods:**

We used latent transition analysis (LTA) to assess the MIL profiles of Chinese university students and to relate their experiences of meaning to their wellbeing. Meaning in Life Questionnaire, Brief Symptom Inventory, and the Mental Health Continuum Short Form were applied. In total, 317 students from five universities in Hong Kong participated in the survey at two time points 9 months apart.

**Results:**

The LTA identified three distinct profiles among the participants: meaning-oriented, bewildered, and indifferent. The LTA mover–stayer model revealed the relative stability of the students’ MIL profiles over 9 months. Specifically, the indifferent profile group was the most unstable, with a stability of 66.6%, suggesting that a significant portion of students in this group changed profiles. Conversely, the bewildered profile group had the greatest number of movers (64.8%), indicating a higher degree of flux within this group as well. Regarding the adaptive outcomes associated with each profile, results showed that students in the meaning-oriented profile group demonstrated the most adaptive outcomes, evidenced by the highest wellbeing scores and the lowest anxiety and depression scores among all the students.

**Conclusion:**

These findings provide insights into MIL profiles and how they change among Chinese university students. We also identified a relatively adaptive profile. Overall, these findings have practical implications and can contribute to advancing research on mental health and meaning.

## 1 Introduction

The fast-changing nature of the economy, technology, and society is creating growing challenges for young adults globally. Worse still, almost two thirds of youth experience significant adverse events, regardless of where in the world they reside (Carlson et al., 2020). Among adverse childhood experiences, childhood maltreatment (CM) is one concerning problem. The mental health of vulnerable youth is affected profoundly by the developmental crises and trauma inflicted by CM (Fu and Underwood, 2015). In this context, the construct of meaning in life (MIL) has received growing attention alongside burgeoning interest in positive traits and psychological strengths (Ryan and Deci, 2001; Seligman and Csikszentmihalyi, 2000). MIL refers to individuals’ ability to understand their purpose in life and the significance of their existence (Steger et al., 2006). It helps youth establish a stable identity, build intimate relationships, and be productive. It also plays a crucial role in coping with developmental crises and restoring wellbeing in the face of trauma. Thus, it is valuable to investigate the important role of MIL among university students (Bouckenooghe et al., 2022).

Most MIL studies have used the variable-centered approach, which assumes that all respondents belong to the same population and that the effects of MIL apply equally to all of them (e.g., Li et al., 2021). However, this assumption obscures the unique variance explained by specific combinations of MIL dimensions. In contrast to variable-oriented methods, which emphasize the directions and strengths of correlations among variables, person-oriented approaches focus on identifying various patterns among multiple variables based on the similarities of characteristics within a common sample (Li and Cheng, 2022). Thus, we can categorize the sample into heterogeneous subgroups and investigate how the dimensions interact within individuals as well as examine group differences in mental health (e.g., anxiety, depression, and wellbeing; Lin and Shek, 2021).

Considering the malleable nature of MIL, we adopted a longitudinal design to explore whether the students in one subgroup may move to another subgroup over time using latent transition analysis (LTA). Examining MIL transition is of particular interest for understanding young adults’ development of meaning. We thus contribute to meaning theory by verifying the constellations of MIL and describing the mover–stayer probabilities. Our study can thus serve as a stepping-stone toward more theoretical work in meaning theory.

### 1.1 Meaning in life and its dimensions

Frankl (1963) defined MIL as the “will-to-meaning” and proposed that humans possess the essential motive to determine the unique meaning in their lives. Steger et al. (2006) further differentiated two distinct dimensions of MIL—presence of meaning (POM) and search for meaning (SFM). POM concerns the degree to which individuals perceive their lives as significant and meaningful, while SFM reflects the extent to which people actively and intentionally engage in the search of MIL. Specifically, POM emphasizes the experience of meaning, whereas SFM focuses on the process of meaning exploration (Lin and Shek, 2021).

Recently, Zhang et al. (2016) added two important aspects, namely need for meaning (NFM) and meaning confusion (MC), to supplement the original dimensions of MIL (i.e., POM and SFM). NFM refers to peoples’ innate drive to find meaning in their lives (Frankl, 1963), while MC concerns the extent to which one feels confused about MIL or how to attain a meaningful life. These two additional aspects extend the scope of MIL, and they are distinguished from POM and SFM. The convergent and discriminant validities of the two aspects have been well supported (Zhang et al., 2016). In this study, we integrated the four dimensions into one model (i.e., POM, SFM, NFM, and MC) to further explore MIL profiles.

### 1.2 Exploring meaning-in-life profiles with latent transition analysis

Most studies have investigated MIL using the variable-centered approach. Although interaction analysis can examine combinations of MIL dimensions in such an approach, it cannot detect the distinct subgroups that exhibit unique MIL configurations. In contrast, the person-oriented approach is a typological methodology used to explore how different dimensions interact within individuals (Dezutter et al., 2014). As MIL is a multidimensional construct, it is valuable to adopt the person-centered method to capture the complex nature of MIL.

A few studies have confirmed several MIL profiles, consistent with Marcia (1966) ego-identity status theory. This theory proposes that resolving one’s self-identity is the most crucial task within the developmental stage of university students. Finding meaning offers direction in life, such as helping such students decide what they want to engage in and the identity they want to establish for themselves (Bronk, 2011; Burrow and Hill, 2011). Corresponding to Marcia (1966) identity status as identity commitment and crisis engagement, it is possible to redefine the two MIL dimensions, namely POM as “meaning commitment” and SFM as “meaning exploration” (Steger et al., 2006). Correspondingly, two studies identified four types of MIL configurations: (1) meaning achievement, with high POM and SFM; (2) meaning moratorium, with low POM but high SFM; (3) meaning foreclosure, with high POM but low SFM; and (4) meaning diffusion, with low POM and SFM (Li and Cheng, 2022; Lin and Shek, 2021). Another study found only three clusters with the absence of the meaning diffusion profile among Polish college students (Krok, 2018). On the contrary, two studies yielded five clusters, identifying one additional “undifferentiated cluster” with scores for POM and SFM at the average level among the United States (Dezutter et al., 2014) and Romania (Negru-Subtirica et al., 2017) samples. Similarly, using the four additional aspects of MIL, Zhang et al. (2016) reported five configurations of MIL that echo previous findings.

Although certain evidence has been established, several limitations of the literature make further research necessary. First, all of the studies discussed adopted cluster analysis to explore the MIL profiles, entailing an empirical description of the correlated attributes from a clustering algorithm. With advancement in statistics methods, latent profile analysis (LPA), which can be seen as a special and better type of cluster analysis, has become increasingly popular in recent years. LPA assumes that people can be typed with varying degrees of probabilities into categories (subpopulations). It is a model-based technique that provides fit statistics, which is a key advantage over heuristic cluster approaches (e.g., k-means clustering; Spurk et al., 2020). Second, the studies all had cross-sectional designs. In contrast, a longitudinal design would be more appropriate for capturing the dynamic nature of MIL. LTA is an extension of LPA that uses longitudinal data to identify transitions between latent profiles over time. In this sense, we consider class membership as being a dynamic developmental stage (Abarda et al., 2020). It serves as an elegant solution for studying heterogeneous changes in longitudinal data. Third, in terms of MIL complexity, the use of a four-dimensional model can provide a more comprehensive picture of MIL profiles. So far, no studies have examined MIL with a four-dimensional model using LTA. To address these limitations, we explored whether specific MIL profiles can be distinguished among Chinese university students and evaluated the transition probabilities among MIL profiles over time using a four-dimensional model.

### 1.3 Meaning in life and mental health

MIL is the essence of human experience and is critical to mental health (Hicks and Routledge, 2013). The literature has demonstrated that POM is negatively associated with mood disorders, such as depression and anxiety (Chen et al., 2021). Furthermore, POM has been consistently found to predict happiness, wellbeing, and quality of life across different age groups from diverse cultures (e.g., Dezutter et al., 2014; Li and Cheng, 2022). For university students in particular, a sense of MIL is an important psychological resource to facilitate positive developmental outcomes (Cotton Bronk et al., 2009). Nevertheless, the relationships between SFM and positive outcomes have remained inconclusive. For instance, SFM has often been found to be related to undesirable outcomes, such as anxiety and depression (e.g., Steger et al., 2008a,b). Interestingly, however, people with high levels of POM and SFM have reported greater life satisfaction, more happiness, and less depression (Park et al., 2010).

The utilization of a variable-oriented approach can be one of the reasons for the inconclusive relationships among POM, SFM, and wellbeing (Dezutter et al., 2014). As mentioned earlier, such an approach is not able to demonstrate how multiple variables are configured within individuals (De Fruyt et al., 2002). In contrast, a person-centered approach has the advantage of being able to identify distinct groups of people within a common sample. Thus far, limited studies have provided information on how different MIL profiles are associated with psychological functioning longitudinally, especially with a four-dimensional model. Thus, we investigated the relationships between MIL profiles and mental health outcomes to determine whether an “optimal” MIL profile exists.

### 1.4 Present study

The primary aim of this study is to identify distinct MIL profiles among Chinese university students. We employed Latent Profile Analysis (LPA) to uncover specific configurations of meaning dimensions within individuals. Drawing on prior research that integrates identity theories (commitment and exploration; Marcia, 1966) and meaning theories (POM and SFM; Steger et al., 2006), we hypothesize that: (1) Profile Identification: Specific MIL profiles will emerge from the data; however, the exact number of profiles is not predetermined. (2) Longitudinal Stability: LTA will be used to examine the stability of these MIL profiles over time. We aim to explore whether individuals maintain their initial MIL profile or transition to different profiles. (3) Mental Health Outcomes: We will investigate the relationship between MIL profiles and mental health indicators, including anxiety, depression, and wellbeing. This analysis will provide a more comprehensive understanding of the implications of different MIL profiles.

## 2 Methods

### 2.1 Participants and procedure

Students from five major universities in Hong Kong completed the online self-reported survey at two time points 9 months apart (in September 2019 and June 2020). Their universities’ Institutional Review Boards approved the study before commencement (No. H002654). The inclusion criteria for the study were identical at each time point: (1) aged over 18 years old and (2) enrolled as an undergraduate student. The first survey had 317 participants (26.2% male) and the second had 302 participants (26.8% male). The attrition rate was 4.04%. As participation was voluntary, the reasons for attrition during T2 could not be tracked. Feedback from participants of T2 indicated that its timing, coinciding with end-of-year exams, led to some students being unable to participate due to increased academic pressure. The participants had a mean age of 19.53 (SD = 1.28) years. The participants came from 5 years of study, with 28.07% in Year 1, 22.40% in Year 2, 21.45% in Year 3, 13.56% in Year 4, and 14.51% in Year 5.

The students were invited to the survey via the mass emailing system of each of the five universities sampled. The survey was prefaced with an introduction to the study. Informed consent was collected on the first page before they start the survey. It stated that participation was voluntary as well as assured the students that the information provided was not connected to their grades and that their data would be kept confidential and would only be accessed by the research staff. The students were instructed to answer the survey questions based on what immediately came to mind when reading them. The survey took about 20 min to complete on average.

### 2.2 Measures

#### 2.2.1 Meaning in life

The Meaning in Life Questionnaire (Steger et al., 2006) was used to assess the presence of and search for meaning, with five items per dimension. Two additional dimensions, MC and NFM, were taken from the Extended Meaning in Life Questionnaire (Zhang et al., 2016). Sample items include, “My life has a clear sense of purpose” (POM), “I am looking for something that makes my life feel meaningful” (SFM), “I think a life without meaning is pointless” (NFM), and “I do not know how to obtain meaning in my life” (MC). The participants were required to rate each item on a 7-point Likert scale ranging from 1 (not true at all) to 7 (definitely true). Research has shown that these subscales have good reliability and validity in Chinese culture and among Chinese adolescents (Datu and Yuen, 2022), and that they can be combined to comprehensively measure MIL. The Cronbach’s alpha values of all subscales at both time points are listed in Table 1, ranging from satisfactory to excellent.

**Table 1:** Descriptive statistics and correlations of variables (*N* = 302).

	1	2	3	4	5	6	7	8	9	10	11	12	13	14	15	16
1 NFM T1	1															
2 NFM T2	0.40**	1														
3 MC T1	−0.07	- 0.14*	1													
4 MC T2	−0.04	0.12*	0.62**	1												
5 PFM T1	0.20**	0.15*	−0.44**	−0.31**	1											
6 PFM T2	0.13*	0.54**	−0.30**	−0.20**	0.42**	1										
7 SFM T1	0.68**	0.30**	0.04	0.03	0.24**	0.14*	1									
8 SFM T2	0.33**	0.76**	−0.02	0.17**	0.13*	0.51**	0.34**	1								
9 CM T1	−0.14**	−0.12*	0.28**	0.16**	−0.24**	−0.14*	−0.08	−0.07	1							
10 CM T2	−0.10*	−0.20**	0.28**	0.23**	−0.19**	−0.28**	−0.04	−0.14*	0.60**	1						
11 DEP T1	0.02	−0.14*	0.54**	0.42**	−0.15*	−0.21**	0.06	−0.01	0.25**	0.26**	1					
12 DEP T2	0.01	0.11	0.36**	0.57**	−0.18**	−0.12*	−0.02	0.16**	0.18**	0.32**	0.49**	1				
13 ANX T1	0.13*	−0.08	0.35**	0.26**	−0.04	−0.12*	0.12*	0.01	0.16**	0.17**	0.66**	0.40**	1			
14 ANX T2	0.05	0.19**	0.20**	0.35**	−0.14*	0.04	0.01	0.16**	0.09	0.25**	0.36**	0.70**	0.46**	1		
15 WB T1	0.27**	0.21**	−0.46**	−0.34**	0.47**	0.30**	0.23**	0.13*	−0.30**	−0.24**	−0.44**	−0.40**	−0.37**	−0.29**	1	
16 WB T2	0.15**	0.24**	−0.37**	−0.45**	0.38**	0.44**	0.18**	0.21**	−0.23**	−0.29**	−0.39**	−0.52**	−0.29**	−0.36**	0.64**	1
Mean	5.62	5.47	4.05	3.89	4.30	4.21	5.27	5.14	2.52	2.51	2.56	2.52	2.87	2.80	2.93	2.67
SD	0.89	1.08	1.42	1.42	0.83	0.96	0.88	1.05	0.46	0.48	0.78	0.80	0.97	0.97	0.89	0.92
α	0.88	0.94	0.89	0.79	0.86	0.92	0.87	0.80	0.73	0.74	0.90	0.89	0.91	0.88	0.86	0.84

*p < 0.05, **p < 0.01. NFM, Need for meaning; MC, Meaning confusion; PFM, Presence of meaning; SFM, Search for meaning; CM, child maltreatment; DEP, Depression; ANX, Anxiety; WB, Wellbeing; T1, Time 1; T2, Time 2.

#### 2.2.2 Depression and general anxiety

The six-item depression and three-item general anxiety subscales in the Brief Symptom Inventory 18 (Andreu et al., 2008) were used to assess depressive and anxiety symptoms among the participants. The participants responded to each item based on how much that problem had distressed or bothered them during the previous 7 days, using a 5-point Likert scale ranging from 0 (did not apply to me at all) to 4 (applied to me very much or most of the time). Sample items include “Feeling no interest in things” (depression) and “Nervousness or shakiness inside” (anxiety). The Chinese version of this scale has been validated and widely used (Huang et al., 2019).

#### 2.2.3 Wellbeing

Wellbeing was measured using the Mental Health Continuum Short Form (MHC-SF) (Keyes et al., 2008). It measures positive mental health and comprises 14 items representing various feelings of wellbeing (i.e., emotional, psychological, and social wellbeing). The respondents were asked to rate their wellbeing over the previous month ranging from 1 (never) to 6 (every day). The MHC-SF has shown good psychometric properties in the literature (Lamers et al., 2011). The items were translated into Chinese and back translated into English to ensure comparability.

### 2.3 Data management and analyses

First, descriptive analysis and correlation analysis on the data were performed by SPSS 25.0. Then, LPA was conducted to determine a set of latent classes of the participants based on their responses to different dimensions of MIL. Once LPA solutions were selected for the two time points, LTA was applied to the data to determine the probability of profile membership at Time 2 (T2) given profile membership at Time 1 (T1) (Chung et al., 2005). LTA integrates autoregressive (a variable predicting itself in the future) modeling (Nylund et al., 2006) to examine group membership over time with transition outcomes. It maps how students’ MIL moves between these groups, providing probability estimates for both group membership and transition, thereby precisely describing the students’ MIL transition.

For LTA, fit was assessed using five fit indices: two likelihood ratio tests and three information criterion indices. The LMR-A and BLRT both provide a test of whether there is a statistically significant improvement for an addition of one more class to the model (Nylund, 2007). Akaike (1987) information criterion (AIC), the Bayesian information criterion (BIC) (Schwarz, 1978), and the sample size adjusted BIC (ABIC) model are each a selection criterion, lower values of which indicate the preferred model. As a post-hoc evaluation of group separation, an entropy criterion was used. High entropy (> 0.8) indicates a high accuracy rate of classification (Lubke and Muthén, 2007). In addition to these indices and fit criteria, decisions regarding the optimal number of classes were guided by the relative size of the classes and their theoretical meaningfulness.

Finally, analysis of variance (ANOVA) was conducted to assess the variance explained by the finalized profiles for each subscale. Differences in wellbeing, depression, and anxiety among the participants in different profile groups were explored. All latent analyses were conducted using Mplus 7.0 (Muthén et al., 2017).

## 3 Results

### 3.1 Descriptive statistics and correlations of all variables

The descriptive statistics and correlations of all variables are shown in Table 1. At both time points, NFM was positively correlated with wellbeing, but negatively correlated with CM. MC was positively correlated with depression, anxiety, and CM, but negatively correlated with wellbeing. POM was positively correlated with wellbeing, but negatively correlated with depression and CM. SFM was positively correlated with wellbeing. Finally, NFM and SFM (T1 and T2) were positively correlated with anxiety (T1 and T2), respectively.

### 3.2 Latent profile analysis

Both time points were tested for two to five latent group profiles. The fit results for the two waves are presented in Table 2. The BIC supported a three-group solution for both time points. The BIC never reached a lowest point and the elbow (i.e., the last relatively large decrease in the BIC value) was used as a guide. In addition, the LMR-A and BLRT tests approached non-significance (*p* > 0.05) and entropy was maintained at a relatively high value (> 0.80), suggesting good separation for the groups (Sorgente et al., 2019). According to the statistical model fit indices and criteria, the three-profile solution was preferred.

**Table 2:** Goodness of fit indices for different latent profile analysis models (*N* = 317 for Time 1 and *N* = 302 for Time 2).

Model	AIC	BIC	ABIC	Entropy	LMR-A	BLRT	Profile prevalence					
							1	2	3	4	5	6
2-Profile	3518.03	3566.89	3525.66	0.922	< 0.001	< 0.001	0.124	0.876				
**3-Profile**	**3431.61**	**3499.27**	**3442.18**	**0.840**	**0.002**	**0.002**	**0.117**	**0.700**	**0.183**			
4-Profile	3377.14	3463.60	3390.65	0.799	0.077	0.084	0.218	0.539	0.053	0.189		
5-Profile	3344.41	3449.66	3360.85	0.807	0.045	0.048	0.009	0.101	0.170	0.202	0.517	
6-Profile	3301.06	3425.10	3320.44	0.840	0.420	0.430	0.022	0.104	0.457	0.031	0.233	0.151
Time 2												
2-Profile	3233.88	3282.12	3240.89	0.863	< 0.001	< 0.001	0.169	0.831				
**3-Profile**	**3131.61**	**3239.27**	**3228.18**	**0.793**	**0.044**	**0.048**	**0.146**	**0.637**	**0.217**			
4-Profile	3143.72	3229.06	3156.12	0.746	0.423	0.437	0.131	0.033	0.328	0.508		
5-Profile	3120.83	3224.72	3135.92	0.795	0.340	0.347	0.129	0.510	0.311	0.010	0.040	
6-Profile	3099.57	3222.02	3117.36	0.822	0.208	0.215	0.007	0.013	0.128	0.297	0.044	0.511

AIC, Akaike information criterion; BIC, Bayesian information criterion; ABIC, Adjusted Bayesian information criterion; LMR-A, Lo–Mendell–Rubin adjusted likelihood ratio test; BLRT, Bootstrapped likelihood ratio test. Bold values: According to the statistical model fit indices and criteria, the 3-profile solution was preferred.

A two-time-point LPA of configural similarity, including three profiles per time point, was then estimated. This model was then contrasted against a model of structural similarity by constraining the within-profile means on MIL to be equal across time points. Compared with the model of configural similarity, this model resulted in lower AIC and BIC values, thereby supporting the structural similarity of this solution across time points. This model was then contrasted against a model of dispersion similarity in which the within-profile variance of MIL was constrained to be equal across time points. Compared with the model of structural similarity, this LTA resulted in lower values for all information criteria, thus supporting the dispersion similarity of the solution. Finally, we estimated a model of distributional similarity by constraining the size of the latent profiles to be equal across time points. Compared with the model of dispersion similarity, this model resulted in lower AIC, BIC, and ABIC values, thus supporting the distributional similarity of the solution across time points. This model of distributional similarity was illustrated and retained for interpretation and the subsequent stages (Supplementary Table S1).

**Figure 1:**
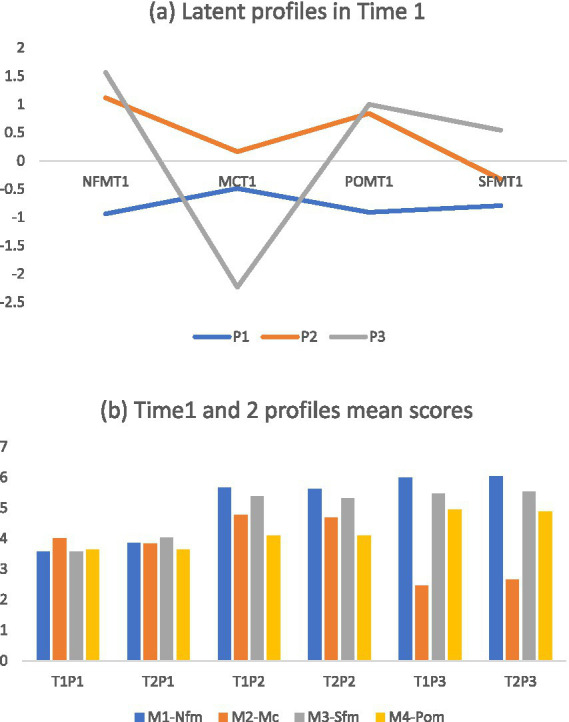
**(A)** Latent profiles based on the four dimensions in Time 1. **(B)** The profiles mean scores in Time 1 and 2. P1: Profile 1 (Indifferent); P2: Profile 2 (Bewildered), P3: Profile 3 (Meaning oriented). M1-NFM, mean score for need for meaning; M2-MC, meaning confusion; SFM, search for meaning; POM, presence of meaning.

One large and two moderate-sized groups were present in both time points. The characteristics of each MIL dimension across the profiles are presented in Figure 1 and Tables 2, 3. The largest group demonstrated the “bewildered profile” (Profile 2; 70.0% for T1 and 63.7% for T2). The participants in this profile group scored highest on MC (4.65 for T1 and T2) and demonstrated a relatively high level of NFM. The second group demonstrated the “meaning-oriented profile” (Profile 3; 18.3% for T1 and 21.7% for T2). Most of the participants in this profile group scored highest on NFM and POM and lowest on MC. The third group demonstrated the “indifferent profile” (Profile 1; 11.7 and 14.6% for T1 and T2). The participants in this profile group scored lowest on NFM, SFM, and POM. They also demonstrated relatively low MC scores. Furthermore, to assess the relative difference of the modeled variables across the three groups for each wave, ANOVAs were conducted. All the ANOVAs resulted in significant differences (Table 3). All variables were significantly different across the three final groups.

**Table 3:** Mean scores and ANOVA on the three latent profiles based on meaning of life.

	Profile 1 Indifferent		Profile 2 Bewildered		Profile 3 Meaning		F	*p*	*Post-hoc* comparison
	Mean	SE	Mean	SE	Mean	SE			
NFM T1	3.50	0.22	5.65	0.06	6.12	0.12	220.00	<0.001	1a < 2b < 3c
MC T1	3.97	0.17	4.65	0.10	2.14	0.18	188.43	<0.001	3a < 1b < 2c
PFM T1	3.53	0.27	5.36	0.06	5.53	0.16	99.05	<0.001	1a < 2b < 3b
SFM T1	3.65	0.16	4.14	0.06	5.05	0.09	54.76	<0.001	1a < 2b < 3c
NFM T2	3.91	0.17	5.61	0.08	6.02	0.15	28.27	<0.001	1a < 2b < 3c
MC T2	3.85	0.23	4.65	0.23	2.76	0.31	39.87	<0.001	3a < 1b < 2c
PFM T2	3.99	0.18	5.30	0.07	5.58	0.12	11.69	<0.001	1a < 2b < 3b
SFM T2	3.63	0.14	4.07	0.13	4.88	0.10	24.36	<0.001	1a < 2b < 3c
N T1/T2	37/45		222/167		58/90				

NFM, Need for meaning; MC, Meaning confusion; PFM, presence of meaning; SFM, search for meaning. In post-hoc comparison, 1 stands for Profile 1; 2 for Profile 2; 3 for Profile 3. a,b,c stand for significant differences among the three groups. Double b shows non-significant differences.

### 3.3 Latent transition analysis

As noted above, this final model of distributional similarity was then converted to an LTA using the manual auxiliary three-step approach. The transition probabilities from this LTA are reported in Table 4. Membership of the meaning-oriented profile group (stability of 95.1%) was the most stable over time. The bewildered profile group demonstrated relative stability over time (81.8%). In contrast, the indifferent profile group (66.6%) was less stable over time than the other profile groups. These findings indicate that profiles characterized by more POM are the most stable.

**Table 4:** Transitions probabilities to time 2 profiles.

	Transition probabilities to time 2 profiles		
Time 1	Profile 1 Indifferent	Profile 2 Bewildered	Profile 3 Meaning oriented
Profile 1	0.666	0.334	0.000
Profile 2	0.108	0.818	0.074
Profile 3	0.049	0.000	0.951

**Figure 2:**
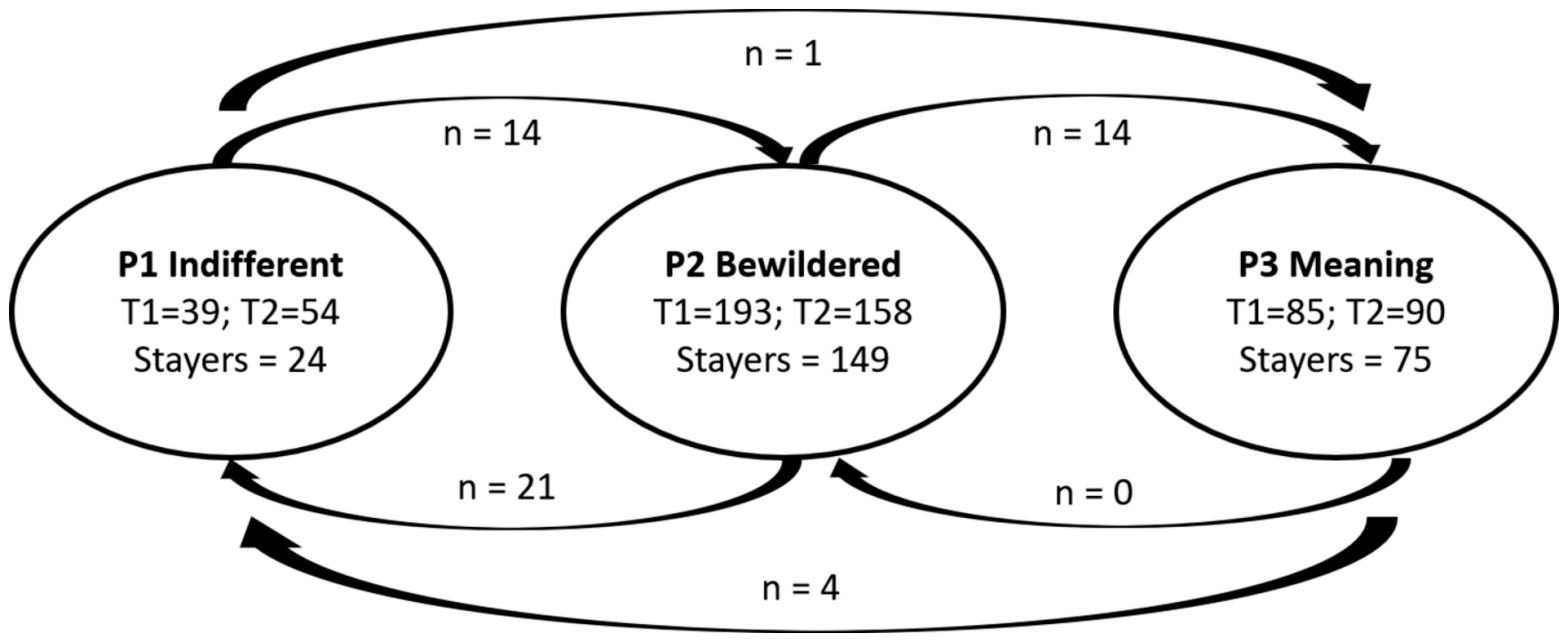
Profiles transition from time 1 to time 2.

In the context of the current analysis, “movers” are individuals whose profile groups change from T1 to T2, whereas “stayers” refer to individuals who remain in their T1 group throughout the study. The pattern of transition between groups is presented in Figure 2, which shows the samples for the mover–stayers. Fourteen students moved from the indifferent to the bewildered profile group. No students moved from the meaning-oriented profile group to the bewildered profile group, but four students moved to the indifferent profile group. The bewildered profile group had the greatest number of movers (35 individual, 64.8%), indicating a higher degree of flux within this group. Specifically, 21 students moved from the bewildered profile group to the indifferent profile group and 14 students moved to the meaning-oriented profile group. Finally, one student moved from the indifferent profile group to the meaning-oriented profile group.

### 3.4 Profile differences in mental health

To test for explanatory similarity, the outcomes were added to the LTA model of distributional similarity described earlier. We first estimated a model in which the within-profile levels of outcomes were freely estimated across the time points. Then, we contrasted this model with another model in which these levels were constrained to equality across time point. As shown in Supplementary Table S1, the latter model resulted in the lowest value for all information criteria when compared with the alternative model, thus the second model was selected. The within-profile means and SDs of each outcome are reported in Table 5.

**Table 5:** Within-time comparisons of profiles on outcomes.

	Profile 1 Indifferent Mean (SD)	Profile 2 Bewildered Mean (SD)	Profile 3 Meaning Mean (SD)	F	Summary of Sig differences
Depression T1	2.333 (0.708)	2.720 (0.753)	2.043 (0.649)	21.610***	3 < 1 < 2
Anxiety T1	2.541 (0.775)	3.003 (0.916)	2.483 (1.094)	9.445***	3 = 1 < 2
Wellbeing T1	2.418 (0.908)	2.786 (0.787)	3.679 (0.703)	37.109***	1 < 2 < 3
DepressionT2	2.599 (0.736)	2.718 (0.619)	2.159 (0.658)	19.772***	3 < 1 < 2
Anxiety T2	2.710 (0.780)	2.914 (0.961)	2.682 (0.892)	5.843**	3 = 1 < 2
Wellbeing T2	2.303 (0.883)	2.450 (0.843)	3.247 (0.784)	31.797***	1 < 2 < 3

**p < 0.01, ***p < 0.001.

These results clearly support the distinct nature of the profiles. The pattern of associations between the profiles and outcomes is also consistent across most outcomes, with the most desirable levels of wellbeing (higher level), depression (lower level), and anxiety (lower level) observed in the meaning-oriented profile group. All pairwise comparisons of wellbeing and depression in T1 and T2 were significant (*p* < 0.05). Anxiety levels followed a similar order across the profiles but demonstrated fewer significant differences. Anxiety levels were highest and undistinguishable in Profile 2, but they were not significantly different between Profiles 1 and 3 at both T1 and T2.

## 4 Discussion

MIL has been widely acknowledged as an important aspect of mental health (Steger et al., 2006). In this study, we aimed to understand university students’ experience of MIL based on four dimensions (i.e., NFM, SFM, MC, and POM) and relate their meaning experience to mental health. We estimated the number of latent groups within a sample of Chinese university students at two time points. Three distinct meaning patterns were identified: bewildered, meaning-oriented, and indifferent meaning profiles. Students in the meaning-oriented profile group were the most stable, whereas the bewildered profile group had the most movers. The students in the meaning-oriented profile group reported more positive wellbeing outcomes than those in the two other groups.

First, using a four-dimensional scale to assess meaning in life (MIL), we identified three distinct profiles among the participants: (1) the indifferent profile, (2) the bewildered profile, and (3) the meaning-oriented profile. This classification contrasts with previous research that utilized the two-dimensional scale (POM and SFM) to identify four groups: (1) meaning achieved (corresponding to the meaning-oriented profile), (2) uncommitted (corresponding to the bewildered profile), (3) foreclosed, and (4) diffused (which aligns with the indifferent profile; Steger et al., 2006). One key difference between our study and earlier research is the absence of the meaning foreclosure group—characterized by high POM and low SFM—in our findings.

The absence of a meaning foreclosure group in this study may be explained by the unique developmental stage that university students typically experience. This period is often marked by significant turbulence and transition, as students confront various academic and existential challenges. University life is a time of exploration, self-discovery, and identity formation, where students must navigate academic pressure, career choices, financial uncertainties, and the complexities of social and intimate relationships. These challenges may prevent students from achieving the high POM with low SFM that would typically characterize a meaning foreclosure group, especially without substantial reflection on their sense of purpose or life goals. Additionally, this may also indicate that university students, despite their challenges, are not as likely to adopt rigid, premature commitments to meaning or purpose. In contrast, they may be more open to exploration and change, which is consistent with the high levels of instability observed in the bewildered profile group. This openness to change and development is a characteristic feature of university students, who are often at a point in their lives where they are beginning to question and refine their beliefs, values, and life goals.

In contrast, our findings indicate that a majority of students (63.7–70.0%) fell into the bewildered profile, characterized by high SFM and low POM. This suggests that many students in this stage are not yet fully engaged in reflecting on or committing to a clear sense of meaning but instead experience a sense of satisfaction or contentment with their current life situation. However, the lack of clarity in their long-term goals and purpose leaves them in a state of flux, indicative of a transitional period where they are still searching for deeper meaning and direction in life. This may reflect the developmental nature of identity formation in late adolescence and early adulthood, where students are often preoccupied with the present moment or short-term goals rather than long-term purpose.

Second, scholars have suggested that people are more likely to demonstrate high SFM with low POM. Interestingly, in the present study, high POM and high SFM were found to coexist in the meaning-oriented profile group. This positive relationship between POM and SFM contrasts with previous findings, which have been negatively correlated among Western samples (Steger et al., 2008a,b). Conceptually, Frankl (1963) considered SFM as a natural and healthy process leading to increased POM, whereas Baumeister (1991) believed SFM to be a response evoked by a crisis of meaning. Nevertheless, findings from Asian contexts have indicated otherwise as high POM along with high SFM (Chen et al., 2021; Steger et al., 2008a,b). For instance, studies have reported positive or non-significant relationships among Chinese and Japanese samples (Chen et al., 2021). Our findings support this positive relationship. Scholars have tended to adopt cognitive style theory and cultural value perspectives to understand this contradiction (Lin and Shek, 2021; Steger et al., 2008a,b). The dialectical or holistic thinking style is widely promoted in Asian cultures, which emphasizes the acceptance and reconciliation of contradiction. Therefore, people from Asia tend to perceive the world as dynamic and embrace contradictions (De Oliveira and Nisbett, 2017). In addition, Asian cultures value continuous efforts to strive for a better and more socially recognized self. Even without confusion or deficiency in meaning, SFM itself represents an ongoing self-improvement tendency of exploring or deepening one’s understanding of life (Lin et al., 2021). Thus, the two seemingly contradictory dimensions of MIL are more likely to co-exist among Asian samples.

Third, in terms of the longitudinal observation of LTA movers-stayer results, in general, more than 82.1% of the students’ MIL profiles remained the same over the 9 months between T1 and T2. The meaning-oriented profile group was the most stable. This result suggest that a person’s sense of meaning is relatively stable and resistant to fluctuations even during turbulent periods of life. This finding aligns with broader theories in psychology that suggest meaning in life can be a stable construct, grounded in deep personal values, even as individuals encounter new challenges or life changes. Although it is not easy for youth to achieve meaning-oriented status, fortunately, once MIL is present, it is relatively stable and easy to maintain (Steger, 2018).

The students in the indifferent and bewildered profile groups were more likely to change their MIL. As the students in the bewildered profile group had the largest proportion of students, the number of movers was the highest in this group. They are still in the process of searching for or solidifying their sense of purpose. The fact that they are more likely to transition between profiles indicates that their experience of meaning is more fluid and subject to change. They may be experiencing shifts in their beliefs, values, and goals, which can lead to fluctuations in their sense of meaning. This can be explained by the idea of cycling through stages during transitions or in response to crisis (e.g., Super et al., 1990). For instance, when students face an existential crisis, such as trying to recover from the loss of a family member or experiencing the uncertainty of the COVID-19 pandemic, they feel the urge to search for MIL.

Moreover, the most significant movement was observed in the indifferent profile group, which suggests that students in this category might be undergoing a more dramatic shift toward either increased or decreased engagement with meaning-making processes. The indifferent profile, characterized by low POM and low SFM, could represent a stage of existential disengagement or indifference, where students are either not actively reflecting on their life purpose or are struggling to connect their life experiences with broader meaning. Over time, some of these students may transition into a more meaning-oriented or bewildered profile as they begin to confront questions of purpose, identity, and future goals, spurred by the challenges of university life.

Finally, our findings echo those of previous studies that MIL is important to human functioning (e.g., Steger, 2018; Zika and Chamberlain, 1992), with the students who perceived their life as meaningful demonstrating better mental health status. The meaning-oriented profile group showed significantly more adaptive outcomes (i.e., lower depression scores and higher wellbeing scores). That is, students who perceived their lives as meaningful demonstrated significantly better mental health outcomes, reinforcing the notion that a sense of purpose and meaning contributes to psychological resilience and wellbeing.

However, an intriguing finding emerged when we examined anxiety levels between the meaning-oriented and indifferent profile groups. Contrary to expectations, the anxiety levels between these two groups were not significantly different, suggesting that the absence of a strong sense of meaning may not directly correlate with higher anxiety. This could be because anxiety in young adults is influenced by a range of factors beyond meaning in life, such as academic pressures, social stressors, or existential concerns related to the transition to adulthood (McLean and Breen, 2018). Students in the indifferent group could be disengaged from the deeper processes of self-reflection or existential inquiry, potentially leading to a kind of passive indifference to life’s meaning, which does not necessarily translate into higher anxiety.

This finding is particularly important because it suggests that a high sense of meaning does not necessarily imply a lack of struggle or anxiety about life’s uncertainties. The meaning-oriented students may be actively searching for meaning, which can be an intense and at times anxiety-provoking process, yet their overall psychological functioning was more adaptive, as reflected in their lower depression scores and higher wellbeing ratings. This aligns with the literature showing that individuals who are actively SFM—though they may experience greater anxiety—are also likely to be more open-minded, curious, and engaged with the world around them (Steger, 2018; Steger et al., 2008a,b). Nevertheless, the process of SFM, despite the associated anxiety, often fosters greater personal growth, resilience, and a deeper connection to one’s values, goals, and purpose in life. This idea of “meaning making” aligns with the existential perspective, which posits that meaning is not something that is passively received, but rather something that individuals actively create and reconstruct throughout their lives (Yalom, 2020).

## 5 Implications

Our findings offer valuable information and have important practical implications for university students and education sectors. First, since students with higher MIL tend to have more positive mental health outcomes, it would be beneficial to provide students with tools for meaning-making—such as mindfulness practices and narrative approaches. Second, MIL interventions should be tailored to students’ developmental stages. School intervention for MIL from a younger age (e.g., primary and secondary school) could help young people develop a strong foundation for mental health. Third, teacher training for MIL is important for youth development. Educators play a pivotal role in shaping the lives of young people and equipping them with the skills to foster MIL can have a profound impact. By integrating meaning-centered practices, educators can support students’ wellbeing (e.g., Shin and Steger, 2014).

## 6 Limitations and future research directions

The results of this study should be considered for certain study limitations. First, the sample size was relatively small and only included undergraduate students in Hong Kong. Future research should try to recruit more diverse samples, given the very little cross-cultural work done in this field to date. Comparing MIL profiles and their changes in different cultures would be fruitful. Second, only self-report methods were used in this study. Methodologists in the MIL literature has been mostly limited to self-reports. Additional methods, such as experimental manipulation and behavioral observation, could deepen the understanding of MIL. Third, we only used two time points with 9 months apart in this study. The 9-month period may be insufficient to capture the changes in MIL profiles. In future work, three or more time points with longer period could provide more information regarding changes in MIL. Finally, although our attrition rate (4.04%) in this study was acceptable, the academic pressure during the T2 data collection period may have influenced students’ responses, particularly for those experiencing heightened anxiety. This limitation highlights the need for careful consideration of timing in longitudinal studies, as stress factors may impact participation rates and the stability of mental health outcomes over time.

## 7 Conclusion

In conclusion, this study provides important insights into the meaning in life (MIL) profiles of Chinese university students, highlighting both the stability and changes in these profiles over time. Through latent transition analysis, we identified three distinct MIL profiles—meaning-oriented, bewildered, and indifferent—and examined how these profiles relate to wellbeing outcomes. The results suggest that while certain profiles, such as the meaning-oriented group, are associated with more positive mental health outcomes. These findings not only contribute to a deeper understanding of MIL dynamics in a university context but also have practical implications for mental health interventions and the promotion of meaning and wellbeing among students.

## Data Availability

The original contributions presented in the study are included in the article/Supplementary material, further inquiries can be directed to the corresponding author.
