# Supplementary material for: Stability and changes in meaning in life profiles and their impact on mental health among chinese university students: a latent transition analysis
Source: Front Psychol. 2025 Feb 6;16:1529851. doi: 10.3389/fpsyg.2025.1529851 (PMC11840567; doi:10.3389/fpsyg.2025.1529851)
Supplement: Supplementary file 1 [file Table_1.docx]

Supplementary material

Table 1 Results from the longitudinal latent transition analyses (T1, N = 317, T2 (N = 302)

| Model | LL | #fp | Scaling | AIC | BIC | ABIC | Entropy |
| --- | --- | --- | --- | --- | --- | --- | --- |
| Configural Similarity | -3202.923 | 40 | 1.115 | 6485.846 | 6636.202 | 6509.332 | 0.852 |
| Structural Similarity | -3209.526 | 28 | 1.306 | 6475.053 | 6580.302 | 6491.493 | 0.852 |
| Dispersion Similarity | -3211.124 | 24 | 1.358 | 6470.248 | 6560.462 | 6484.340 | 0.851 |
| Distributional Similarity | -3221.662 | 22 | 1.340 | 6467.323 | 6560.019 | 6480.240 | 0.851 |
| Predictive Similarity |  |  |  |  |  |  |  |
| Free relations with predictors | -3147.572 | 24 | 1.318 | 6343.144 | 6433.052 | 6356.932 | 0.831 |
| Equal relations with predictors | -3137.397 | 26 | 1.344 | 6326.794 | 6424.195 | 6341.731 | 0.847 |
| Exploratory Similarity |  |  |  |  |  |  |  |
| Free relations with outcomes | -5353.039 | 70 | 1.590 | 10846.077 | 11109.200 | 10887.177 | 0.844 |
| Equal relations with outcomes | -5348.982 | 70 | 1.121 | 10837.964 | 11101.087 | 10879.064 | 0.861 |
